# Proactive distractor suppression elicited by statistical regularities in visual search

**DOI:** 10.3758/s13423-021-01891-3

**Published:** 2021-02-23

**Authors:** Changrun Huang, Ana Vilotijević, Jan Theeuwes, Mieke Donk

**Affiliations:** 1 Department of Experimental and Applied Psychology, Vrije Universiteit Amsterdam, Van der Boechorststraat 7-9, 1081 BT Amsterdam, The Netherlands; 2 Institute Brain and Behavior (iBBA), Amsterdam, the Netherlands

**Keywords:** Statistical learning, Salience, Visual selection

## Abstract

Irrelevant salient objects may capture our attention and interfere with visual search. Recently, it was shown that distraction by a salient object is reduced when it is presented more frequently at one location than at other locations. The present study investigates whether this reduced distractor interference is the result of proactive spatial suppression, implemented prior to display onset, or reactive suppression, occurring after attention has been directed to that location. Participants were asked to search for a shape singleton in the presence of an irrelevant salient color singleton which was presented more often at one location (the high-probability location) than at all other locations (the low-probability locations). On some trials, instead of the search task, participants performed a probe task, in which they had to detect the offset of a probe dot. The results of the search task replicated previous findings showing reduced distractor interference in trials in which the salient distractor was presented at the high-probability location as compared with the low-probability locations. The probe task showed that reaction times were longer for probes presented at the high-probability location than at the low-probability locations. These results indicate that through statistical learning the location that is likely to contain a distractor is suppressed proactively (i.e., prior to display onset). It suggests that statistical learning modulates the first feed-forward sweep of information processing by deprioritizing locations that are likely to contain a distractor in the spatial priority map.

Imagine driving down the road and focusing on the traffic and road signs, when the red blinker in your car turns on. At first, your attention will be captured by this blinking light, as a salient object tends to draw attention (e.g., Donk & van Zoest, 2008; Itti & Koch, 2001; Theeuwes et al., 1998). However, after noticing that the light is irrelevant, you might continue driving normally without being distracted by the blinker.

This experience is anecdotal. Yet research has shown that observers are in fact able to ignore an irrelevant distractor, in particular when it repeatedly appears at one specific location. It has been shown that observers learn statistical regularities regarding probable distractor locations which in turn leads to a reduced distractor interference (Feldmann-Wüstefeld & Schubö, 2016; Ferrante et al., 2018; Wang & Theeuwes, 2018a, 2018b). In recent experiments, Wang and Theeuwes (2018a, 2018b) employed the classical additional singleton paradigm (Theeuwes, 1991, 1992), in which participants search for a shape singleton target while ignoring a salient task-irrelevant color singleton distractor. Unbeknownst to participants, the probability of the distractor location was manipulated such that the distractor appeared more often in one location (the high-probability location) than in other locations (the low-probability locations). The results showed that reaction times (RTs) were shorter when the distractor was presented in the high-probability location as compared with low-probability locations.

Based on these results, it was argued that after several trials, the high-probability location becomes suppressed through a process of statistical learning, which facilitates target selection. It has been suggested that participants implicitly learn the underlying characteristics of the search display such that the location that is likely to contain a distractor is suppressed relative to all other locations.

There are in principle two ways in which this spatial suppression can be implemented: proactively (prior to display onset) and reactively (after attentional engagement). Traditionally, it was assumed that distractor suppression is *reactive*, resulting from disengaging attention from an attended location, an effect that is reminiscent of inhibition of return (IOR; Klein, 2000). Theeuwes (2010) argued that capture effects in the original additional singleton task may be small or in some condition even absent, not because there was no attentional capture but instead because of very fast disengagement. The original idea was that attention is initially captured (even for the briefest moment) by the salient singleton, and if it turns out not to be the target, it is immediately suppressed. Reactive suppression can explain why, due to statistical learning (Wang & Theeuwes, 2018a), capture is reduced for the location that is likely to contain a distractor relative to other locations. Indeed, it is feasible that following attentional capture, observers have learned to disengage faster from the location that is likely to contain a distractor than from the other locations.

Overall, a large number of studies suggest that distractor suppression can only occur in a reactive manner (e.g., Beck et al., 2018; Humphreys et al., 2004; Lahav et al., 2012; Lahav & Tsal, 2013; Makovski, 2019; Moher & Egeth, 2012; Tsal & Makovski, 2006; Won et al., 2019). For instance, Moher and Egeth (2012) showed that if observers were instructed to ignore a particular distractor feature, target search was slower than in a neutral condition in which no prior feature information regarding the distractor was provided. According to Moher and Egeth (2012), suppression can only occur in a reactive manner, as attention first has to be directed to the location of the feature in order to be able to suppress it (see also Theeuwes, 2010). Importantly, in Moher and Egeth (2012), participants did not possess any (implicit or explicit) knowledge regarding the likely location of the irrelevant distractor. Possibly, the suppression here occurred in a reactive manner because identifying the to-be-ignored distractor feature likely required attention (but see Gaspelin et al., 2015, 2017, for an alternative account). Yet support for the idea that irrelevant distractors are reactively suppressed has also been provided in studies in which there was no need for feature identification (Humphreys et al., 2004; Makovski, 2019; Tsal & Makovski, 2006). Using a prestimulus probe method in combination with a classical flanker task, Tsal and Makovski (2006) showed that when participants knew the locations of upcoming flanker distractors, those locations received more rather than less attentional processing capacity prior to display onset. They concluded that to-be-ignored distractor locations are typically attended, even before display onset, and that subsequent suppression of those locations can only occur afterwards, at a later stage of processing.

Alternatively, it has been proposed that suppression is *proactive* (Wang & Theeuwes, 2018a, 2018b, 2018c)*.* The idea is that through statistical learning, the spatial priority map gets altered such that locations that are likely to contain a distractor compete less for attention than all other locations. This results in a reduced saliency signal for objects presented at this suppressed location (Ferrante et al., 2018; Wang & Theeuwes, 2018a, 2018b, 2018c), which in turn leads to reduced attentional capture for distractors presented at the high-probability relative to the low-probability locations. Critically, proactive location suppression in the priority map is assumed to happen prior to the presentation of the search display, thus preventing attention to be captured by anything presented at that location (Huang et al., 2020; Kong et al., 2020; Wang, van Driel, et al., 2019). Evidence from fMRI studies also suggested the feasibility of proactive suppression. Several studies showed that a cue that induced the anticipation of an upcoming distractor evoked prestimulus neural activity in visual cortex, which can be linked to the process of distractor suppression (Munneke et al., 2011; Ruff & Driver, 2006; Serences et al., 2004). A recent EEG study by Wang, van Driel, et al. (2019) provided converging evidence that this suppression may indeed be proactive. This study shows that there was increased alpha power contralateral to the location that was likely to contain a distractor relative to the ipsilateral location. This increased alpha power, often considered a neural marker of inhibition (Jensen & Mazaheri, 2010), was already present about 1,200 ms, providing neural evidence for proactive inhibition.

The present study investigated whether the reduced distractor interference as observed in a statistical learning paradigm is the result of *proactive* or *reactive* suppression. In the current experiment, in the majority of trials (two thirds), participants performed the additional singleton search task (Theeuwes, 1991, 1992) in which participants searched for a shape singleton (the target) in the presence of a salient color singleton (the distractor). Critically, the probability of the distractor’s appearance in different locations was varied. One of the locations represented a high-probability location, meaning that in 65% of all trials in the search task, the distractor singleton appeared there. In 35% of trials, the distractor singleton appeared equally often at one of the remaining locations (low-probability locations). In a minority of trials (one third) participants were asked to perform a probe task in which they had to detect the offset of a single dot, which could occur equally likely at each of the locations in the display.

We used a probe offset rather than a probe onset method to ensure that there were no sudden luminance onsets in the displays which are known to affect the distribution of attention. This probe offset technique was previously used by Theeuwes and Godijn (2002), and replicated by Folk and Remington (2006). Because there are no abrupt onsets in the display, this technique allows a clear representation of how attention is distributed across the different locations in the display.

It is important to realize that if participants had to detect the offset of the probe (one third of the trials), this was done before the presentation of the search display (see Fig. 1). This implies that the probe task probes the distribution of attention across all locations in the display prior to the presentation of the search display. In other words, it provides a snapshot of how participants prepare for the upcoming search display.

**Fig. 1:**
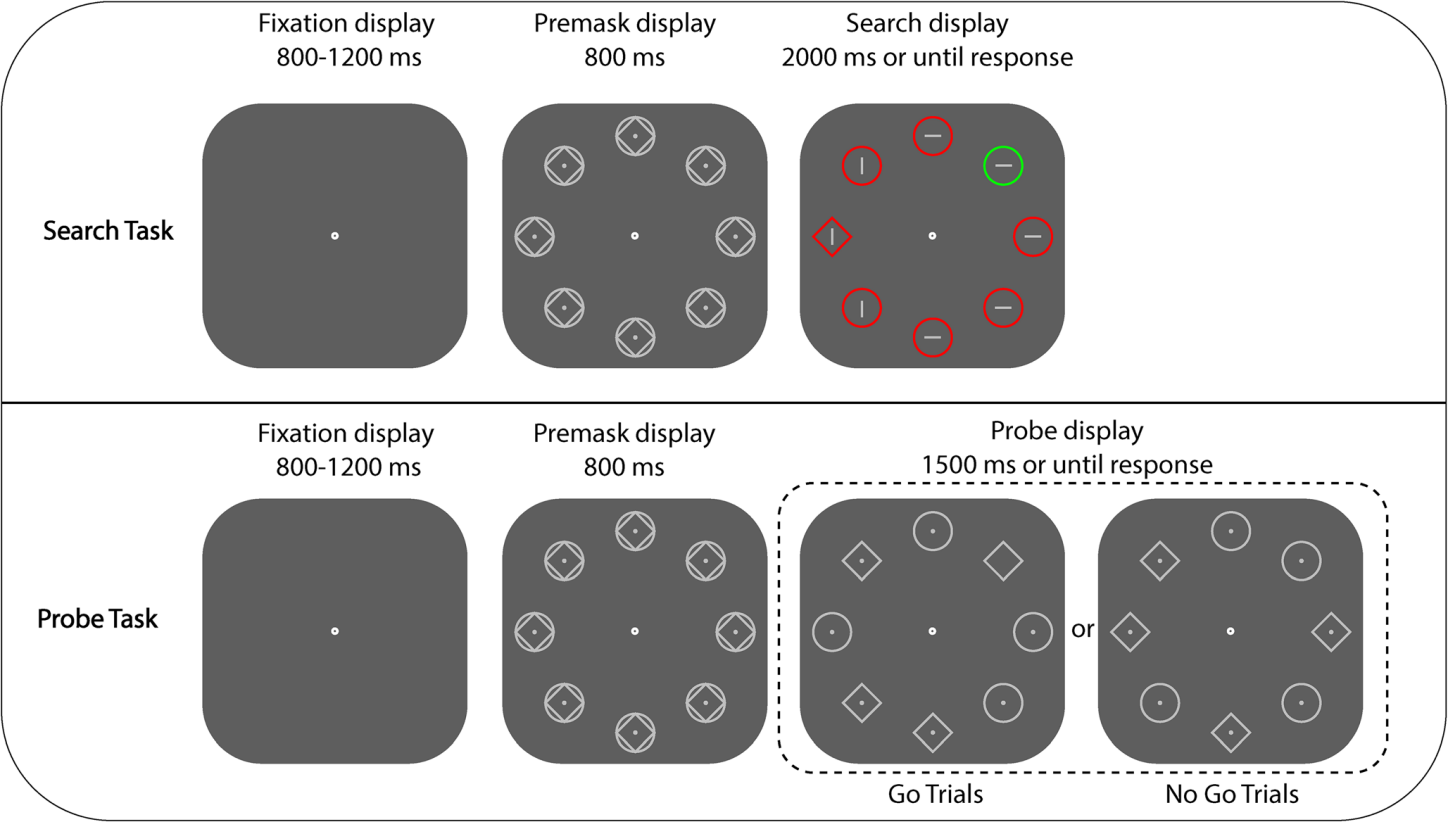
The upper panel–Example of consecutive displays presented in the search task. Participants were asked to search for the target shape singleton (either a diamond among circles or a circle among diamonds) in the presence of an irrelevant distractor color singleton (either a green shape among red shapes or a red shape among green shapes). The distractor singleton was presented more often in the high-probability location than in the low-probability location. The lower panel—Example of consecutive displays presented in the probe task. Participants were asked to indicate the presence of a dot offset (go trials) or refrain from responding (no-go trials). (Color figure online)

The current study tested the various alternative ways of how suppression is implemented. If distractor suppression occurs proactively (Wang, van Driel, et al., 2019), participants are expected to be slower in detecting the probe offset when presented at a high-probability than at a low-probability location, as this one is suppressed. Alternatively, if distractor suppression is reactive, it does not occur prior to the onset of the search display, but only after the high-probability location has been selected. If attentional selection also occurs after display onset (Moher & Egeth, 2012), then one expects that before search display onset, attention is evenly distributed across the display and probe-offset detection performance should be unaffected. If attention is already directed to the high-probability location prior to search display onset (Tsal & Makovski, 2006), to allow later suppression, participants should be faster in detecting a probe offset presented at the high-probability location than at the other locations.

## Method

### Participants

In order to determine the sample size, we conducted a pilot study with a sample of 20 naïve participants (six females, *M*_age_ = 26.4, *SD*_age_ = 5.03), who were recruited via Prolific. The pilot study was identical to the main experiment. The results of the pilot study showed a probe RT difference of 44.54 ms, with larger RTs for detecting probes offset at the high-probability than at the low-probability locations. However, the effect size obtained on the basis of pilot data is usually inflated which may lead to a follow-up bias (Albers & Lakens, 2018). We therefore took half of this effect size (RT difference = 22 ms) as the smallest effect size of interest in an a priori power analysis for our main study. The power analysis (using the *simr* package of Green & MacLeod, 2016) based on the pilot data indicated that a sample size of 40 participants would have a power of 84% (95% CI [81.58, 86.22] in 1000 simulations) to detect a probe RT difference of 22 ms. Considering that online studies as compared with those performed in a lab can yield noisier data as well as larger drop-out rates, we recruited 79 participants (22 females, *M*_age_ = 22.81 years, *SD*_age_ = 3.38 years) via Prolific for the main experiment. All participants received a monetary reward (£5.63) in exchange for 45 minutes of participation. Before the experiment, all participants provided written informed consent.

The experiment was approved by the Ethical Review Committee of the Faculty of Behavioral and Movement Sciences of Vrije Universiteit Amsterdam and was conducted in accordance with the guidelines of the Helsinki Declaration.

### Stimuli and task

The experiment was programmed in JavaScript and run via OSWeb (Mathôt et al., 2012). The link to the experiment as well as demographic questions and instructions were displayed in Qualtrics. The experiment employed a *search* task in two-thirds of trials and a *probe* task in one-third of the trials.

#### Search task

Each search trial started with the presentation of a fixation dot (18 × 18-px)^1^ display lasting for the jittered interval between 800 and 1,200 ms, while the fixation dot remained visible throughout each trial. This was followed by a premask display for 800 ms. The premask display consisted of eight equidistant elements (diamonds surrounded by circles) placed on an imaginary circle with a radius of 224 pixels around the central fixation dot. The size of each element was 92 × 92 pixels. All elements were light gray outlines (RGB: 192/192/192) and each contained a light gray dot in the middle (10 × 10 px; RGB: 192/192/192). Following the presentation of the premask display, the search display was presented consisting of one shape singleton (the target), one color singleton (the distractor), and six other elements (see Fig. 1). All elements had the same shape (diamond or circle), except for the target, which was either a diamond (among circles) or a circle (among diamonds) with equal probability. All elements had the same color (red or green), except for the distractor which was colored either red (among green elements) or green (among red elements) with equal probability. Each of the elements contained a gray line (36 × 4 px; RGB: 192/192/192), which was equally likely horizontally or vertically oriented. All elements were superimposed on a dark gray background (RGB: 94/94/94). The distractor could appear in all locations. However, one of the locations represented a high-probability location, meaning that in 65% of all trials of the search task, the distractor singleton appeared at that position. In the remaining search-task trials (35% of the trials), the distractor singleton appeared equally often at one of the other locations (the low-probability locations). The target was equally likely presented at one of the locations unoccupied by the distractor. The search display lasted for 2,000 ms or until a response was given (see Fig. 1). Participants were instructed to search for a uniquely shaped element (the target) and indicate the orientation of the line it contained by pressing either the “up” or “left” arrow key for vertical or horizontal orientations, respectively. Prior to the experiment, participants were instructed to respond as fast and accurately as possible.

#### Probe task

The probe task was similar to the search task except that immediately after the presentation of the premask display, a probe display was presented for 1,500 ms. The probe display consisted of four circles and four diamonds randomly distributed within the visual array. In 20% of all probe-task trials (no-go trials), each shape contained a light gray dot in the middle, similar to the premask display. In 80% of all probe-trials (go trials), one dot was missing in the probe display, creating a probe offset at that location relative to the premask display. The probe offset could occur equally likely at each of the eight locations. Participants were instructed to press the “A” key as fast as possible in trials with a probe offset (go trials) and withhold responding in trials without (no-go trials). Both accuracy and reaction times were emphasized in this task.

### Design and procedure

Before the beginning of the experiment, participants were asked to answer two demographic questions (age and gender). The entire experiment consisted of a practice phase followed by an experimental phase. During the practice phase, participants received written and iconic instructions with regard to the search task followed by a first practice block consisting of 50 search trials which were randomly selected from the full pool of experimental search trials. Next, participants received written and iconic instructions about the probe task, after which the second practice block was presented. This block also consisted of 50 trials but included both search and probe trials which were randomly selected from the full pool of experimental trials. During the practice blocks, participants received auditory (2700 Hz, square waveform) and visual feedback (i.e., the fixation dot turned red for 800 ms) each time they made a mistake so as to ensure that they fully understood both tasks.

The experimental phase consisted of 400 search trials and 200 probe trials. The search trials comprised 260 trials in which the distractor was presented at the high-probability (65%) location and 140 trials in which the distractor was presented at the low-probability (35%) location. The position of the high-probability location was constant for each individual participant, but was counterbalanced across participants. The target was equally often presented at one of the seven locations unoccupied by the distractor. Distractor color (red or green), target shape (circle or diamond), and line orientation within the target singleton (horizontal or vertical) were counterbalanced across the search trials. The probe trials comprised 40 no-go and 160 go trials in which probe offsets occurred equally often at each of eight locations. The search trials and the probe trials were randomly intermixed with the constraint that two probe trails could not be presented in sequence and that the very first trial of the first experimental block always consisted of a search trial. Subsequently, these trials were separated into five blocks with 120 trials each. During the experimental phase, participants only received written feedback (average RTs and the percentage correct) after each block of trials. After the experiment, participants’ awareness regarding the statistical regularities of the distractor location was assessed. They were asked if they were aware of the high-probability location of the distractor, and if so, they were asked to mark which location that was and to express their confidence in the answer on a seven-point Likert scale (1 = *very doubtful*, 7 = *very confident*).

### Data-analysis

#### Outliers removal

The RT data of both the search task and the probe task were processed offline using a custom script written in Python. Participants whose mean accuracy for either the search task or the probe task was below 70% were excluded. Participants whose mean RT (collapsed across conditions) for either the search task or the probe task was above or below ±2.5 standard deviation of the overall mean RT were excluded. For the analyses of mean RTs in the search trials, incorrect and both fast (<200 ms) and slow (>2 000 ms) responses were excluded. Also, for the analyses of mean RTs in the probe task, we excluded go trials where responses were incorrect or faster than 200 ms.

We analyzed the accuracy data with the generalized linear mixed models (GLMMs) and RTs with the linear mixed models (LMMs) using the *lme4* package (Bates et al., 2015) in R (R Core Team, 2020). Mixed-effects models are favored over repeated-measures analysis of variance (RM ANOVA) for the reasons that the data are treated at the observation level (i.e., trial) and therefore retain richer information than participant-wise aggregated data. This approach provides more power and can deal with unbalanced design and missing data (Brysbaert & Stevens, 2018). For the search task, the accuracy data and RTs were analyzed separately with distractor location (coded as 1 = high-probability location, 0 = low-probability location) as a fixed effect. We included by-participants random intercepts and by-participants random slopes for distractor location. To control the probable advantages of the specific physical location of the target in a particular trial, we included physical target location (0~7; dummy-coded) as an additional fixed effect. As a search trial could be either preceded by another search trial or a probe trial, the target could appear at a location that was occupied by the target in the preceding search trials or by the offset of the probe dot in the preceding probe trials. To control the inter-trial location priming, we included target-target location (same, different; dummy coded) and probe-target location (same, different; dummy coded) as fixed effects. To track the emergence of suppression over time, RTs and accuracy were separately analyzed as a function of the order of the search trials using the SMART method (van Leeuwen et al., 2019). The trials from practice blocks were also included in this analysis as they had the same regularity settings as the experimental blocks. Note that for the RTs analysis, search trials with incorrect responses were excluded, which left around 480 search trials. A moving Gaussian window (step size = 1 and σ = 15) was used between the first and the 480th trial to create weighted smoothed time series. We used cluster-based permutation testing to control for multiple comparisons. This procedure was repeated a thousand times for each participant (1,000 permutations; see van Leeuwen et al., 2019, for further details).

For the probe task, RTs were entered into the LMMs as a dependent variable with distractor location (coded as 1 = high-probability location, 0 = low-probability location) as a fixed effect. By-participants random intercepts and by-participants random slopes for distractor location were included as a random effect. The physical probe location in a particular trial (0~7; dummy-coded) was included as an additional fixed effect to control participants’ potential bias toward a specific location. To control for intertrial location priming, target–probe location (same, different; dummy-coded) was also entered as a fixed effect. The *p* values were obtained by the likelihood ratio test for all model comparisons in which the model with the fixed effect was compared against the model without. Previous studies (Wang & Theeuwes, 2018a, 2018b, 2018c) suggested that the spatial regularities of the distractor might induce distributed suppression centered around the high-probability location. This suppression gradually decreases as the spatial distance from the high-probability location increases. To examine this effect, we defined a factor distance as the distance between the probe dot location and the high-probability location: distance (Dist-0, Dist-1, Dist-2, Dist-3, Dist-4). Note that Dist-0 represents the high-probability location. Next, we entered RTs into the LMMs as a dependent variable, with distance (Dist-0, Dist-1, Dist-2, Dist-3, Dist-4; dummy-coded) as the fixed effect of interest. The other fixed effects included physical probe location (0~7; dummy-coded) and target–probe location priming (same, different; dummy-coded). By-participants random intercepts and by-participants random slopes for distance were included as random effects. For comparisons within factors, the degrees of freedom were estimated by Satterthwaite approximation, and the *p* values were obtained from the *lmerTest* package (Kuznetsova et al., 2017). The estimate (β) of the fixed effect was provided as the measure of the effect size.

## Results

In total, 19 participants were excluded from the main study on the basis of the predetermined exclusion criteria. The analyses were performed on the data of the remaining 60 participants.

### Search task

**Fig. 2:**
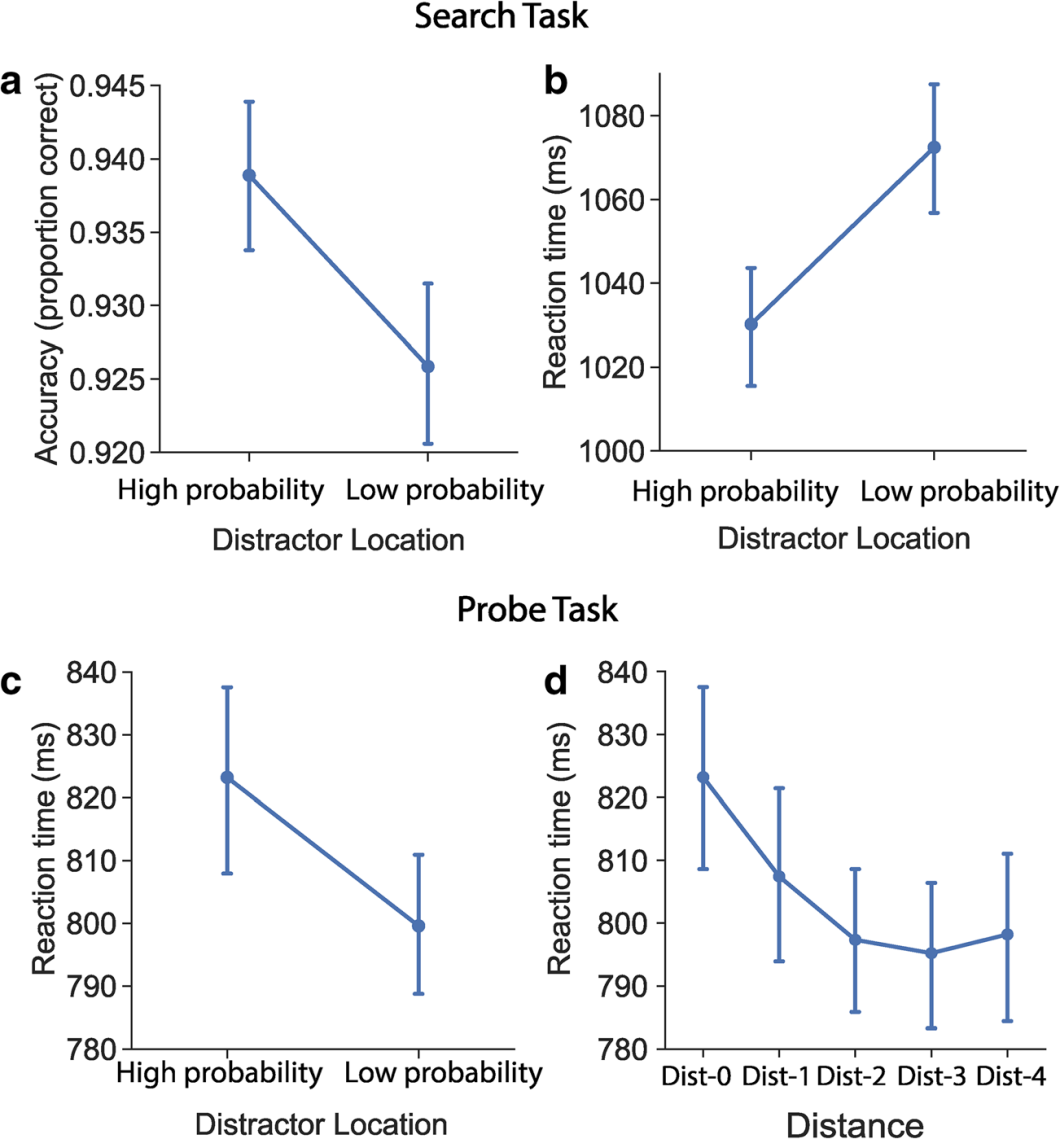
Mean accuracy (**a**) and mean reaction times (**b**) as a function of distractor location (high probability, low probability) in the search task. Mean reaction times as a function of (**c**) distractor location (high probability, low probability) and as a function of (**d**) distance (Dist-0, Dist-1, Dist-2, Dist-3, Dist-4) in the probe task. Error bars denote ±1 *SE*_mean_

**Fig. 3:**
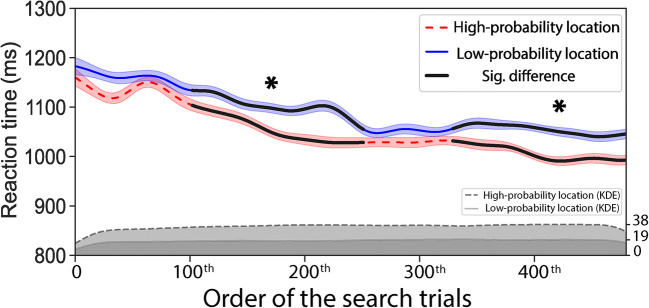
Reaction time smoothed as a function of the order of search trials for when the distractor was presented in the high-probability location (red dashed line) and in the low-probability location (blue solid line). Asterisks indicate significant clusters after cluster-based permutation testing. The shaded area around the lines shows the weighted 95% confidence intervals. The kernel density estimations below the smoothed time series show the estimated trial number per millisecond. (Color figure online)

Mean accuracy and mean reaction times as a function of distractor location are shown in Fig. 2a–b. Both the GLMMs analysis on the accuracy data and the LMMs analysis on the RTs revealed a significant fixed effect for distractor location, accuracy: χ^2^(1) = 14.45, *p* < .001; RTs: χ^2^(1) = 56.49, *p* < .001. The results show higher accuracy (β = .27, *SE* = 0.07, *z* = 4.01, *p* < .001) and shorter RTs (β = −42.74, *SE* = 4.41, *t*(60) = −9.70, *p* < .001), when the distractor was presented at the high-probability location as compared with the low-probability location indicating that attentional capture by the salient distractor was reduced when it was presented at the high probability location. Figure 3 shows the development of reaction time across search trials. As indicated by two clusters (100th ~ 250th trial, 340th ~ 480th trial) in Fig. 3, participants were faster to find the target when the distractor was presented at the high-probability location than at the low-probability location (*p* < .001), suggesting that participants learned to suppress the high-probability location from around the 100th trial on. No differences were found in the time courses of the accuracy data.

### Probe task

Overall, participants performed well in the probe task. Both the false-alarm rates in the no-go trials (*M* = 0.08, *SD* = 0.06) and the miss rates in the go trials (high-probability location: *M* = 0.024, *SD* = 0.042; low-probability locations: *M* = 0.020, *SD* = 0.022) were very low. Figure 2c shows the mean reaction times as a function of distractor location in the probe task. Note that only the go trials with correct responses were included in the RTs analysis. The LMMs analysis on the RTs revealed a significant fixed effect for distractor location, χ^2^(1) = 14.98, *p* < .001. Probe-offset detection was slower for offsets at the high-probability location than at the low-probability location (β = 28.82, *SE* = 6.98, *t*(59.8) = 4.13, *p* < .001). To investigate the spatial distribution of the suppression, the mean reaction times as a function of distance were plotted in Fig. 2d. The LMMs analysis showed a significant fixed effect for distance, χ^2^(4) = 15.62, *p* = .004. The post hoc comparisons with Bonferroni correction indicated that the detection of probe was slower at Dist-0 (i.e., the high-probability location) than at Dist-2 (β = 30.24, *SE* = 7.84, *t*(64.6) = 3.86, *p* = .003), Dist-3 (β = 35.42, *SE* = 9.13, *t*(61) = 3.88, *p* = .003), and Dist-4 (β = 35.77, *SE* = 9.66, *t*(59.7) = 3.70, *p* = .005). No differences were found between Dist-0 and Dist-1 (β = 17.20, *SE* = 6.51, *t*(59.7) = 2.64, *p* = .11), and between any of the other distances (all *p*s > 0.26). Together these results suggest that the strength of suppression decreases with increasing distance from the high-probability location.

### Awareness test

Twenty-three out of 60 participants indicated that they were aware of the high-probability distractor location during the experiment (*M*_confidence_ = 4.39, *SD*_confidence_ = 1.20). However, only eight of them correctly indicated the position of the high-probability location. Removing those eight participants had no significant influence on the results.

## Discussion

The present study shows that distraction by a salient object is reduced when the distractor is presented more often in one location than in other locations. The findings of the probe dot task provide unequivocal evidence that this reduction in capture is due to proactive suppression, as participants were slower to respond to a probe when it was presented at the high-probability location relative to the low-probability locations. Furthermore, our findings also suggest that the suppression was maximal at the high-probability location and gradually decreased along with the increment of the distance to this location. As the probe was presented before search display onset, suppression of the high probability location must already have been in place before the search display was presented. We assume that through statistical learning the more likely distractor location competes less for attention in the spatial priority map than all other locations. Specifically, we assume that prior to display onset the location within the spatial priority map representing the high probability location is suppressed relative to all other locations (Ferrante et al., 2018; Huang et al., 2020; Kong et al., 2020; Wang, Samara, et al., 2019; Wang & Theeuwes, 2018a, 2018b, 2018c; Wang, van Driel, et al., 2019).

In addition to proactive suppression, suppression can also be applied reactively (i.e., suppression that is feature-based and is applied later in time to the salient singleton feature; e.g., Failing, Feldmann-Wüstefeld, et al., 2019a). Even though it is possible that in the current study, feature-based reactive suppression played a role in affecting RTs in search trials, it should be noted that feature-based reactive suppression could not have played a role in probe trials. The reason for this conclusion is simple: the probe task reveals that the location is suppressed before the search display comes on. In fact, in probe trials, the salient feature of the singleton distractor is never presented (only the premask display), so it is impossible to explain the suppression of the probe in terms of a feature-based reactive suppression mechanism. Note that in the current study proactive suppression is applied to the premask display, and as such one could argue that suppression is, in some sense, object-based. Whether one would also obtain suppression when no premask display would have been presented is unclear, but it is unlikely that the spatial priority map is object-agnostic and that spatial suppression can be applied to empty spaces. It is also possible that the premask display is needed to retrieve the memory traces of the display which in turn generates proactive location-specific suppression.

The suppression of the high-probability location emerged after participants were exposed to around 100 search trials. Although it is appealing to compare whether the emergence of suppression across time is different between the aware and unaware group, such an analysis is problematic in the current study because of insufficient power due to the small number of aware participants (*N* = 8). Note, however, that the removal of the 8 aware participants in the current study did not significantly change the results.

Note that the current proactive location suppression account is different from the signal suppression hypothesis as proposed by Gaspelin et al. (2019; Gaspelin et al., 2015, 2017). Gaspelin et al. (2019; Gaspelin et al., 2015, 2017) also claim that locations containing irrelevant salient distractors can be suppressed. Yet they claim that suppression is feature-based and occurs immediately after display onset. That is, when observers are repeatedly presented with displays containing a to-be-ignored feature value, this feature value becomes proactively inhibited leading to rapid suppression of the activity it generates in the priority map, which in turn prevents attentional capture. According to this view, a location becomes suppressed only after the presentation of the search display. This is unlike the current findings where suppression occurs before display onset.

It is important to note that due to the design of the experiment which required a high probability distractor location, the target was presented much less often at this location than at all other locations. This could imply that the effect reported here (and in equivalent studies as, for example, Wang & Theeuwes, 2018a, 2018b, 2018c) is not due to the distractor being presented more often at the high-probability location (resulting in suppression) but simply the result of the target being presented less often at this location. This specific issue was investigated by Failing, Wang, et al. (2019b). They showed no evidence for spatial suppression when one location was less likely to contain a target. They concluded that in these types of experiments, the recurrent presentation of a distractor in one specific location leads to attentional suppression of that location through a mechanism that is unaffected by any regularities regarding the target position (see also Experiment 2 of Huang et al., 2020).

The current findings are also inconsistent with a reactive suppression mechanism (Moher & Egeth, 2012; Tsal & Makovski, 2006). Reactive distractor suppression implies that a distractor can be suppressed, but only after it has been attended. If the distractor location in the search trials would have been suppressed reactively, there would have been no reason for attention to be diverted away from the high-probability location in the probe trials. It is important to note that in most studies that provide evidence for reactive suppression, observers were instructed to ignore a particular feature and not a location (as in the current study). For instance, Moher and Egeth (2012) showed that when observers were cued with regard to a specific distractor feature (such as the color “red”), they responded slower and made more errors as compared with when the cue was uninformative in this respect. To account for these findings, they proposed that observers first attend to a precued distractor feature in order to successfully inhibit its location afterwards. It is important to note that in Moher and Egeth (2012), there were no statistical regularities in the distractor location, rendering proactive location suppression impossible. In contrast, the regularities of distractor location in the current study could be picked up through statistical learning, and subsequently lead to sustained changes in the spatial priority map, biasing attention away from the expected distractor location.

It is interesting to note that the present results are very different from those reported by Tsal and Makovski (2006; Humphreys et al., 2004; Lahav et al., 2012; Lahav & Tsal, 2013; Makovski, 2019). Tsal and Makovski (2006) had participants perform a classical flanker task (Eriksen & Eriksen, 1974), in which they were asked to report a centrally presented target while ignoring two diagonally arranged flanking distractors. To examine the allocation of attention prior to the flanker task, on some trials (20%), a temporal order judgment task was presented instead. Relying on the finding that an attended stimulus is typically perceived to appear before an unattended one (Stelmach & Herdman, 1991), Tsal and Makovski asked participants to decide which one of two simultaneously presented dots, one at an expected distractor location and the other at another location, appeared first. The results showed that the dot presented at the distractor location was perceived to appear prior to the dot presented at the other location, suggesting that more attention was allocated to the expected distractor location. Tsal and Makovski concluded that if a location (or any other stimulus attribute) needs to be ignored, it receives more rather than less attentional processing capacity prior to display onset (see also Makovski, 2019). These findings suggest that if people have prior explicit knowledge regarding a likely distractor location, attention might actually be directed to that location rather than diverted away from it, denoted as the attentional white bear phenomenon. Even though the tasks employed in Tsal and Makovski (2006) and in our study were quite different, one interesting notion may be considered. It is possible that if participants have explicit knowledge about the locations they are supposed to ignore, they cannot help attending it (as in Makovski, 2019), whereas if participants learn implicitly that a location is likely to contain a distractor (as in the current study), proactive suppression may occur.

In summary, the present findings show that statistical learning may result in the proactive suppression of a location that is likely to contain a distractor. We claim that through statistical learning, the spatial priority map is shaped in such a way that the location within this map competes less for attention than all other locations. This highlights the plasticity within the spatial priority map such that selection is optimally adapted to the learned regularities present in the environment.

## Footnotes

1 All dimensions are expressed under the resolution of 1,024 × 768 px.
